# Attenuation of murine antigen-induced arthritis by treatment with a decoy oligodeoxynucleotide inhibiting signal transducer and activator of transcription-1 (STAT-1)

**DOI:** 10.1186/ar1869

**Published:** 2005-12-30

**Authors:** Marion Hückel, Uta Schurigt, Andreas H Wagner, Renate Stöckigt, Peter K Petrow, Klaus Thoss, Mieczyslaw Gajda, Steffen Henzgen, Markus Hecker, Rolf Bräuer

**Affiliations:** 1 Institute of Pathology, Friedrich Schiller University, Jena, Germany; 2 Institute of Physiology and Pathophysiology, Ruprecht Karls University, Heidelberg, Germany; 3 Institute of Pathology, Dietrich Bonhoeffer Clinical Centre, Neubrandenburg, Germany

## Abstract

The transcription factor STAT-1 (signal transducer and activator of transcription-1) plays a pivotal role in the expression of inflammatory gene products involved in the pathogenesis of arthritis such as various cytokines and the CD40/CD40 ligand (CD40/CD40L) receptor-ligand dyad. The therapeutic efficacy of a synthetic decoy oligodeoxynucleotide (ODN) binding and neutralizing STAT-1 was tested in murine antigen-induced arthritis (AIA) as a model for human rheumatoid arthritis (RA). The STAT-1 decoy ODN was injected intra-articularly in methylated bovine serum albumin (mBSA)-immunized mice 4 h before arthritis induction. Arthritis was evaluated by joint swelling measurement and histological evaluation and compared to treatment with mutant control ODN. Serum levels of pro-inflammatory cytokines, mBSA-specific antibodies and auto-antibodies against matrix constituents were assessed by enzyme-linked immunosorbent assay (ELISA). The transcription factor neutralizing efficacy of the STAT-1 decoy ODN was verified *in vitro *in cultured synoviocytes and macrophages. Single administration of STAT-1 decoy ODN dose-dependently suppressed joint swelling and histological signs of acute and chronic arthritis. Delayed-type hypersensitivity (DTH) reaction, serum levels of interleukin-6 (IL-6) and anti-proteoglycan IgG titres were significantly reduced in STAT-1 decoy ODN-treated mice, whereas mBSA, collagen type I and type II specific immunoglobulins were not significantly affected. Intra-articular administration of an anti-CD40L (anti-CD154) antibody was similarly effective. Electrophoretic mobility shift analysis (EMSA) of nuclear extracts from synoviocytes incubated with the STAT-1 decoy ODN *in vitro *revealed an inhibitory effect on STAT-1. Furthermore, the STAT-1 decoy ODN inhibited the expression of CD40 mRNA in stimulated macrophages. The beneficial effects of the STAT-1 decoy ODN in experimental arthritis presumably mediated in part by affecting CD40 signalling in macrophages may provide the basis for a novel treatment of human RA.

## Indroduction

Human rheumatoid arthritis (RA) is a chronic systemic disorder of unknown aetiology, characterized by intimal lining layer hyperplasia, infiltration of the sublining area by macrophages, T and B lymphocytes, plasma cells and other inflammatory cells as well as progressive destruction of joint structures [1,2]. Despite the uncertainty about its aetiology, RA is thought to be an immune-mediated disease promoting inflammation and tissue destruction. Besides the pro-inflammatory cytokines tumour necrosis factor (TNF)-α, interleukin (IL)-1β and IL-6, which mainly derive from macrophages, a dominant T-helper (Th)1-response is associated with the disease, which is characterized by an imbalance of interferon (IFN)-γ over IL-4 [3,4].

IFN-γ as well as many other cytokines from Th cells regulate gene expression and cellular activation, proliferation and differentiation by means of the janus kinase/signal transducer and activator of transcription (JAK/STAT) pathway [5,6]. The binding of these cytokines to their receptors activates a distinct pair of JAK molecules, which are responsible for the phosphorylation and activation of latent cytoplasmic STAT molecules. After phosphorylation on tyrosine or serine residues, STAT molecules homo- or heterodimerize through a SH2-mediated interaction and subsequently translocate to the nucleus, where they can activate the transcription of selective effector genes.

There is some evidence that STAT-1, predominantly induced by IFN-γ, is involved in RA [7,8]. IL-6, highly abundant in the synovial fluid, was identified as the major STAT-3-activating factor in monocytes, but it was also able to activate STAT-1 in other synovial fluid cells [9]. Moreover, elevated levels of STAT-1 protein in phosphorylated and unphosphorylated forms were detected in the synovial tissue of RA patients [10] and the increased expression of STAT-1-dependent genes correlates with the high inflammatory activity of RA patients [8]. These include lymphocyte and antigen-presenting cell genes as well as genes encoding activation markers, transcription factors, signalling molecules, chemokines and chemokine/cytokine receptors. Of particular importance, STAT-1 also regulates CD40 transcription in different cells, either by direct binding of STAT-1 to the γ-activated sequence (GAS) element in the CD40 promoter or by inducing the *de novo *synthesis of the transcription factor interferon regulatory factor-1 (IRF-1) [11,12], which induces not only CD40 but also other mediators such as inducible nitric oxide synthase (iNOS). Thus, as well as inducing mediators governing synovial inflammation, STAT-1 can additionally promote inflammatory processes through the interaction of CD40 with the CD40 ligand (CD40L). However, the activation and/or increased expression of not just STAT-1, but also of STAT-3, STAT-4 and STAT-6 has been demonstrated in RA synovial tissue *in vivo *[13-15].

Activated (tyrosine phosphorylated) STAT dimers bind to two types of DNA motifs: IFN-stimulated response elements or GAS elements [16]. In this study, we have investigated the *in vivo *efficacy of a synthetic decoy oligodeoxynucleotide (ODN) with high similarity to the consensus sequence of STAT-1-binding GAS elements (according to the TRANSFAC databases [17]), intending to trap activated STAT-1 molecules and thus inhibit the transcription of many inflammation-associated genes. As STAT-6, which is activated by IL-4, exerts a very different DNA sequence binding specificity [18], the possibly advantageous anti-inflammatory gene regulation by this Th2 cytokine was preserved in our approach. We used murine antigen-induced arthritis (AIA), as a Th1-mediated experimental arthritis model [19], which is characterized by local and systemic activation of macrophages, [20] as well as synovial fibroblasts [21].

## Materials and methods

### Decoy oligodeoxynucleotide technique

Double-stranded ODNs were prepared from complementary single-stranded phosphorothioate-bonded ODNs obtained from Eurogentec (Cologne, Germany) by melting at 95°C for 5 minutes, followed by a cool-down phase of 3 to 4 h at ambient temperature. The efficiency of the hybridization reaction was verified with 2.5% agarose gel electrophoresis and usually found to exceed 95%. The sequences of the single-stranded ODNs were (underlined letters denote phosphorothioate-bonded bases):

STAT-1 consensus decoy ODN: 5'-catgttatgcatattcctgtaagtg-3';

STAT mutant control ODN: 5'-catgttatgcagaccgtagtaagtg-3'.

The final concentration of ODNs dissolved in TEN buffer (10 mM Tris-HCl, 1 mM EDTA, 150 mM NaCl, pH 7.5) was 0.4 mM. On the basis of previous electrophoretic mobility shift analysis (EMSA) and reverse transcription (RT)-PCR analyses, the maximal effective concentration and optimal pre-incubation time for the decoy ODN in cultured cells were determined to be 10 μM and 4 h, respectively [22]. Decoy ODNs enter target cells without using any cationic lipid or liposomal complex and are protected by phosphorothioate modification against degradation, thus preventing gene transcription for at least 72 h. Using a mutant control ODN lacking the specificity for the DNA consensus binding GAS elements abolished this inhibitory effect, thus demonstrating the specificity of the decoy ODN technique [23-25].

### Animals

Female C57BL/6 mice, 8 to 10 weeks of age, were purchased from Charles River (Sulzfeld, Germany). They were kept under standard conditions in a 12 h/12 h light/dark cycle and fed with standard pellets (Altromin #1326, Lage, Germany) and water *ad libitum*. All animal studies were approved by the governmental commission for animal protection.

### Immunization, arthritis induction and monitoring

Mice were immunized subcutaneously and in the tail root with 100 μg methylated BSA (mBSA; Sigma, Deisenhofen, Germany) in 50 μl saline emulsified in an equal volume of complete Freund's adjuvant (Sigma), which was adjusted to 2 mg/ml of heat-killed *Mycobacterium tuberculosis *(strain H37RA; Difco, Detroit, MI, USA), and intraperitoneally with 5 × 10^8 ^heat-inactivated *Bordetella pertussis *(Chiron Behring, Marburg, Germany) on days -21 and -14. Arthritis was elicited on day 0 by sterile injection of 100 μg mBSA in 25 μl saline into the right knee joint cavity, while the left knee joint remained untreated. Both knee joint diameters were measured before and at various time-points after arthritis induction using an Oditest vernier calliper (Kroeplin Längenmesstechnik, Schlüchtern, Germany). Joint swelling was expressed as the difference in diameter (mm) between the right (arthritic) and left (control) knee joint.

### Treatment

The immunized animals received an intra-articular injection (25 μl) of different concentrations of STAT-1 decoy ODN (0.05, 0.25, 1.25 or 10.0 nmol, each group n = 10) 4 h before arthritis induction (intra-articular injection of mBSA) into the same knee joint. The animals were sacrificed and prepared for histological and immunological evaluation at day 14 of arthritis. In a second series of experiments, groups of immunized mice (n = 10) received an intra-articular injection of 25 μl (0.25 nmol) of STAT-1 consensus decoy ODN, STAT mutant control ODN, or TEN buffer alone (control group) 4 h before arthritis induction. Three days (acute phase of arthritis) or fourteen days later (chronic phase of arthritis) animals were prepared for histological and immunological evaluation. Animals that were held until the chronic phase of AIA were tested on day 7 for delayed-type hypersensitivity (DTH) reaction.

To determine the effect of CD40, immunized animals received an intra-articular injection of 2.5 or 10 μg of an antibody against CD40L (anti-CD154; clone TRAP1, BD Bioscience Pharmingen, Heidelberg, Germany) in 25 μl TEN buffer 4 h before arthritis induction.

### Delayed-type hypersensitivity

For assessment of the DTH reaction, 5 μg mBSA dissolved in 10 μl saline were injected intradermally into the pinna of the right ear on day 7 after arthritis induction. The ear thickness was measured before and 48 h after challenge using the Oditest calliper. Swelling was expressed as the difference (mm) between the ear thickness before and after antigen injection.

### Histology and grading of arthritis

Mice were anaesthetised by ether inhalation on day 3 (acute phase) or day 14 (chronic phase) of arthritis and sacrificed by cervical dislocation. Both knee joints were removed *in toto*, skinned, fixed in 4.5% phosphate-buffered formalin, decalcified in EDTA, embedded in paraffin, cut into 5 μm thick frontal sections, and stained with hematoxylin-eosin for microscopic examination. Sections were examined by two independent observers (PKP and SH) and graded blindly using a semiquantitative score from 0 to 3 (0, no; 1, mild; 2, moderate; 3, severe alterations) for the extent of: synovial lining layer hyperplasia and infiltration of leukocytes into synovial membrane/joint space (both summarized as inflammation); and pannus formation and necrosis/erosion of cartilage (both summarized as destruction). The final arthritis score was evaluated for each animal by calculating the sum of the values for inflammation and destruction (maximal evaluation grade = 12) as described elsewhere [20].

### Cytokine analyses

After cervical dislocation, sera were collected by total bleeding from the carotid artery, clotting at 4°C and centrifugation. Aliquots were stored at -80°C until further analysis. Analyses of murine IL-1β, IL-6 and TNF-α were performed on serum samples, collected at day three and day 14 post arthritis induction, with commercially available ELISA kits according to the manufacturer's instructions (R&D Systems, Wiesbaden, Germany). The sensitivity of the assays was 5 pg/ml for murine TNF-α and 3 pg/ml for IL-1β and IL-6.

### Determination of serum antibodies by ELISA

Specific IgGs to mBSA, native collagen type I and type II, as well as cartilage proteoglycans were measured by ELISA in serum samples collected at day 14 of arthritis as described elsewhere [26]. Briefly, 96-well microplates (Greiner Bio One, Frickenhausen, Germany) were coated with antigen (10 μg/ml mBSA, collagen type I or type II or proteoglycans) overnight. After washing, plates were incubated with serially diluted serum samples and the amount of bound IgG was determined using anti-mouse IgG-peroxidase conjugate (ICN, Eschwege, Germany) and ortho-phenylendiamine (Sigma) as substrate. Extinction was measured at 492 nm against 620 nm with an ELISA reader (Tecan, Crailsheim, Germany).

For determination of the mBSA-specific isotypes IgG1, IgG2a and IgG2b, alkaline phosphatase (AP)-labelled anti-mouse antibodies were used. The following antibodies were applied: anti-mIgG1-AP, anti-mIgG2a-AP (Southern Biotechnology Associates, Birmingham, AL, USA); anti-mIgG2b-AP (R12-3) (BD Bioscience Pharmingen); and standards mIgG1 (MOPC-31c), mIgG2a and mIgG2b (Sigma). After incubation with AP-labelled antibody and thoroughly rinsing the AP substrate (26 μg p-nitrophenylphosphate (Serva, Heidelberg, Germany) in 100 μl 0.05 M Tris buffer, pH 4.8, containing 20 μg magnesium chloride) was added. The reaction was stopped by addition of 5.3 mg sodium carbonate dissolved in 100 μl distilled water, and absorbance was read at 405/620 nm with the microplate reader. Concentrations were expressed as optical density.

### Efficacy of STAT-1 decoy oligodeoxynucleotide on CD40 expression in macrophages *in vitro*

Murine peritoneal RAW264.7 macrophages (American Type Culture Collection, Rockville, MD; ATCC # TIB-71) were cultured in RPMI 1640 (Gibco BRL, Gaithersburg, USA) supplemented with 50 U/ml penicillin (Invitrogen, Carlsbad, CA, USA), 50 μg/ml streptomycin (Invitrogen), 10 U/ml nystatin (Invitrogen) and containing 10% FCS (Gibco BRL) at 37°C in 5% CO_2_-enriched air. The effect of the STAT-1 consensus decoy ODN (10 μM, 4 h pre-incubation) on CD40 mRNA expression was evaluated by adding lipopolysaccharide (LPS) from *Escherichia coli *serotype 026:B6 (1 μg/ml; Sigma) and murine rIFN-γ (500 U/ml; R&D Systems) to the culture medium (12 h incubation time). Total RNA isolation, reverse transcription and PCR for CD40 and elongation factor-2 cDNA were performed essentially as described previously [27]. Amplification of elongation factor-2 served as an internal standard (house-keeping gene).

### Cell preparation/*in vitro *assays

Efficacy of the STAT-1 decoy ODN was additionally tested in synoviocytes *in vitro*. Synoviocytes were obtained through explant cultures from murine synovial tissue dissected from arthritic knee joints. The synoviocyte preparation consists mainly of fibroblast-like cells, but also of some macrophage-like cells. The culture was maintained in complete medium (DMEM, 10 mM HEPES, 1 mM pyruvate, 2 mM glutamine; Gibco BRL), 100 U/ml penicillin (Jenapharm, Jena, Germany), 0.1 mg/ml streptomycin (Gruenenthal, Stolberg, Germany) and supplemented with 20% FCS (Gibco) for seven days in tissue culture plates in a 5% CO_2 _atmosphere at 37°C with a daily medium exchange. Synoviocytes emerged from explanted synovium within seven days. Confluent synoviocytes were removed following trypsin/EDTA (Gibco) treatment and subcultured in complete DMEM supplemented with 10% FCS. Synoviocytes were used for experiments at passage 3–5. Cells were cultured in 6-well plates at a density of 2 × 10^5 ^cells per well, washed and incubated with 10 μM of the STAT-1 decoy ODN or the mutant control ODN. After four hours of preincubation, cells were either stimulated with 250 U/ml murine rIFN-γ (Life Technologies, Karlsruhe, Germany) or left unstimulated for 30 minutes. Thereafter, cells were washed and harvested with ice-cold phosphate-buffered saline and frozen in liquid nitrogen until preparation of nuclear extracts and detection of STAT proteins by EMSA.

### *In vivo *distribution of internalized decoy oligodeoxynucleotide after injection into the joint space

To visualize the uptake of the decoy ODN *in vivo*, Texas-Red labelled ODN was administered intra-articularly and its distribution was observed using laser scanning microscopy in native histological sections 1, 3 and 6 h after injection.

### Electrophoretic mobility shift analysis

Preparation of nuclear extracts from the cultured synoviocytes and subsequent non-denaturing 4% PAGE were carried out as described [12]. In brief, the double-stranded gel shift oligonucleotides (Santa Cruz Biotechnology, Heidelberg, Germany) for sis-inducible element (SIE) were end-labelled with [γ-^32^P]ATP by using the 5'-end labelling kit from Amersham Pharmacia Biotech (Freiburg, Germany). The specificity of the binding reaction was monitored by performing the assay in parallel with the same samples in the presence of a 100- to 1,000-fold excess of non-labelled oligonucleotides. For supershift analyses, the appropriate gel supershift antibody (Santa Cruz Biotechnology) and nuclear extracts were pre-incubated at ambient temperature for 60 minutes before the EMSA was performed. The sequence of the SIE gel shift ODN corresponds to a mutant (m67) of the SIE of the c-fos promoter [28] and can bind STAT-1/STAT-1 homodimers, STAT-1/STAT-3 heterodimers as well as STAT-3/STAT-3 homodimers [29,30].

### Analysis of STAT-1, STAT-3, CD40, and IRF-1 expression by real time PCR

Knee joints from arthritic and control mice were dissected and skinned. The muscle tissue was removed and the bony parts of the joints were prepared including the joint capsules with synovial tissue. The RNA in the knee joint was stabilized in RNA later (Qiagen, Hilden, Germany). The joints were mechanically disrupted by milling with a dismembrator U (Braun Biotech International, Melsungen, Germany) and dispergation of the tissue powder in TRIzol (Invitrogen) with a Polytron 1200 CL homogenizer (Kinematica, Littau/Luzern, Switzerland). After mechanical disruption and homogenization, the RNA was extracted with TRIzol, following the manufacturer's instructions. The DNase treatment of total RNA and the reverse transcription to cDNA were performed with a DNA free™ Kit (Ambion, Woodward, Austin, TX, USA) and Superscript™ II RNase H reverse transcriptase (Invitrogen), respectively. Semiquantification of STAT-1, STAT-3, CD40 and IRF-1 expression by real time PCR was done using the Rotorgene 2000 instrument (LTF Labortechnik, Wasserburg/Bodensee, Germany). The standard curve was prepared by serial dilution of plasmid DNA (Vector pCR^® ^II TOPO^®^; Invitrogen), containing the cDNA of the analysed gene. All samples to be compared for expression differences were run in the same assay as duplicates together with the standards. The expression of β-actin (for STAT-1 and STAT-3) or GAPDH (for IRF-1 and CD40) served as endogenous control to normalize the differences in the amount of cDNA in each sample. Real time PCR analyses were done using SYBR Green I dye (Sigma) and the enzyme Hot Star Taq (Qiagen). Data were calculated as fold changes in gene expression in mice on day 0 compared with the expression on the other investigated days. The mean value of day 0 was set at 100%. The following primers were used: STAT-1, 5'-tgg agg aat gtt tct gtc cc-3' and 5'-cac atg aag gat gcc cac ta-3' (PCR product size 145 base pairs (bp)); STAT-3, 5'-tca ctt ggg tgg aaa agg ac-3' and 5'-tgg tcg cat cca tga tct ta-3' (PCR product size 129 bp); CD40, 5'-ccc tgg gac ttc atg gta aa-3' and 5'-gca cac atg gag gtc aaa tg-3' (PCR product size 68 bp); IRF-1, 5'-gca aaa cca aga gga agc tg-3' and 5'-cag gta gcc ctg agt ggt gt-3' (PCR product size 113 bp); GAPDH, 5'-gac cac agt cca tgc cat cac tgc-3' and 5'-atg acc ttg ccc aca gcc ttg g-3' (PCR product size 137 bp); β-actin, 5'-cca cag ctg aga ggg aaa tc-3' and 5'-tct cca ggg agg aag agg at-3' (PCR product size 108 bp).

### Statistical analysis

The SPSS 10.0 computer program (SPSS Inc., Chicago, IL, USA) was used for all calculations and statistical evaluations. Results in diagrams were expressed as means ± standard error of mean. The results of control and ODN-treated groups were compared with the non-parametric Mann-Whitney *U* test. A *P *value of ≤0.05 was considered statistically significant.

## Results

### Clinical effects of STAT-1 decoy oligodeoxynucleotide treatment on antigen-induced arthritis

**Figure 1:**
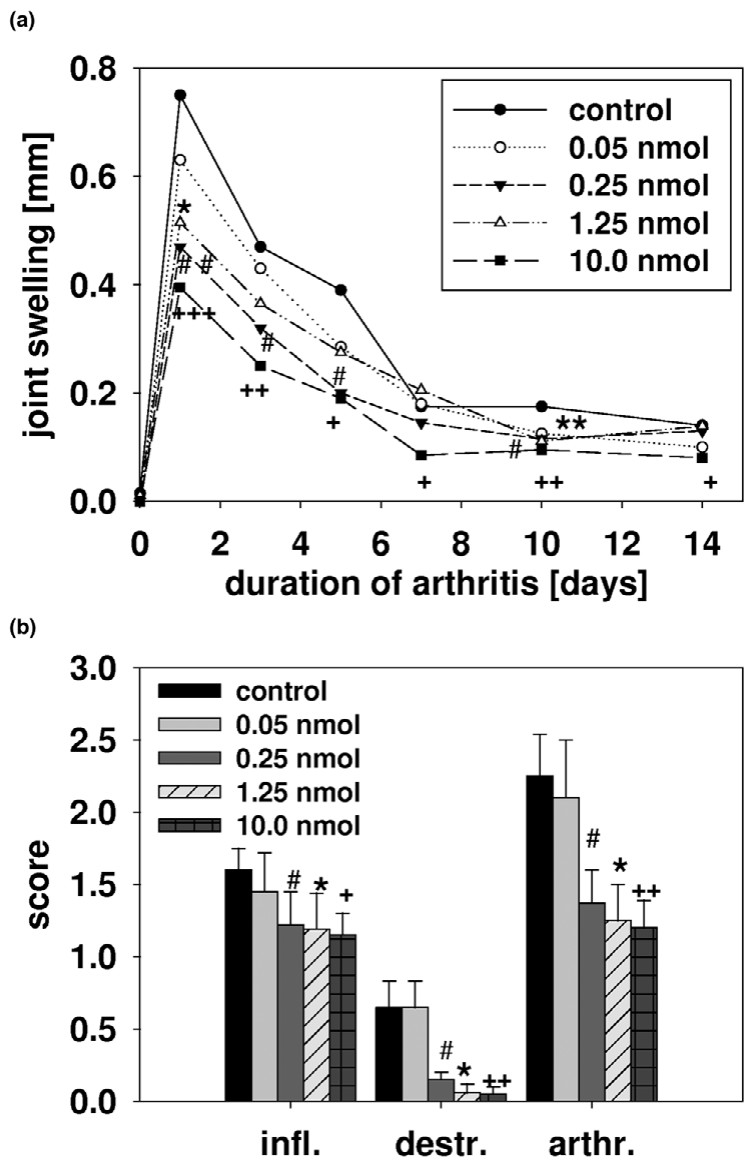
Dose-dependent therapeutic effects of the STAT-1 decoy oligodeoxynucleotide (ODN) on antigen-induced arthritis (AIA). Arthritis was induced in the right knee joint at day 0, 4 h after application of the decoy ODN. **(a) **Effects of the STAT-1 decoy ODN treatment on joint swelling. Joint swelling was assessed as the difference between right (arthritic) and left (untreated) knee joint diameter. **(b) **Histological evaluation of arthritis (day 14 of AIA). Hematoxylin-eosin stained knee sections were evaluated blindly by grading of inflammation (infl.; cell exudate or infiltrate, hyperplasia) and joint destruction (destr.; necrosis, erosion and pannus formation). Total arthritis score (arthr.) is defined as the sum of the inflammation and destruction scores. Each ODN dose represents n = 10;^#^*P *≤ 0.05, ^##^*P *≤ 0.01 (0.25 nmol versus buffer-treated AIA control); *P ≤ 0.05, **P ≤ 0.01, (1.25 nmol versus buffer-treated AIA control); ^+^*P *≤ 0.05, ^++^*P *≤ 0.01, ^+++^*P *≤ 0.001 (10 nmol versus buffer-treated AIA control).

**Figure 2:**
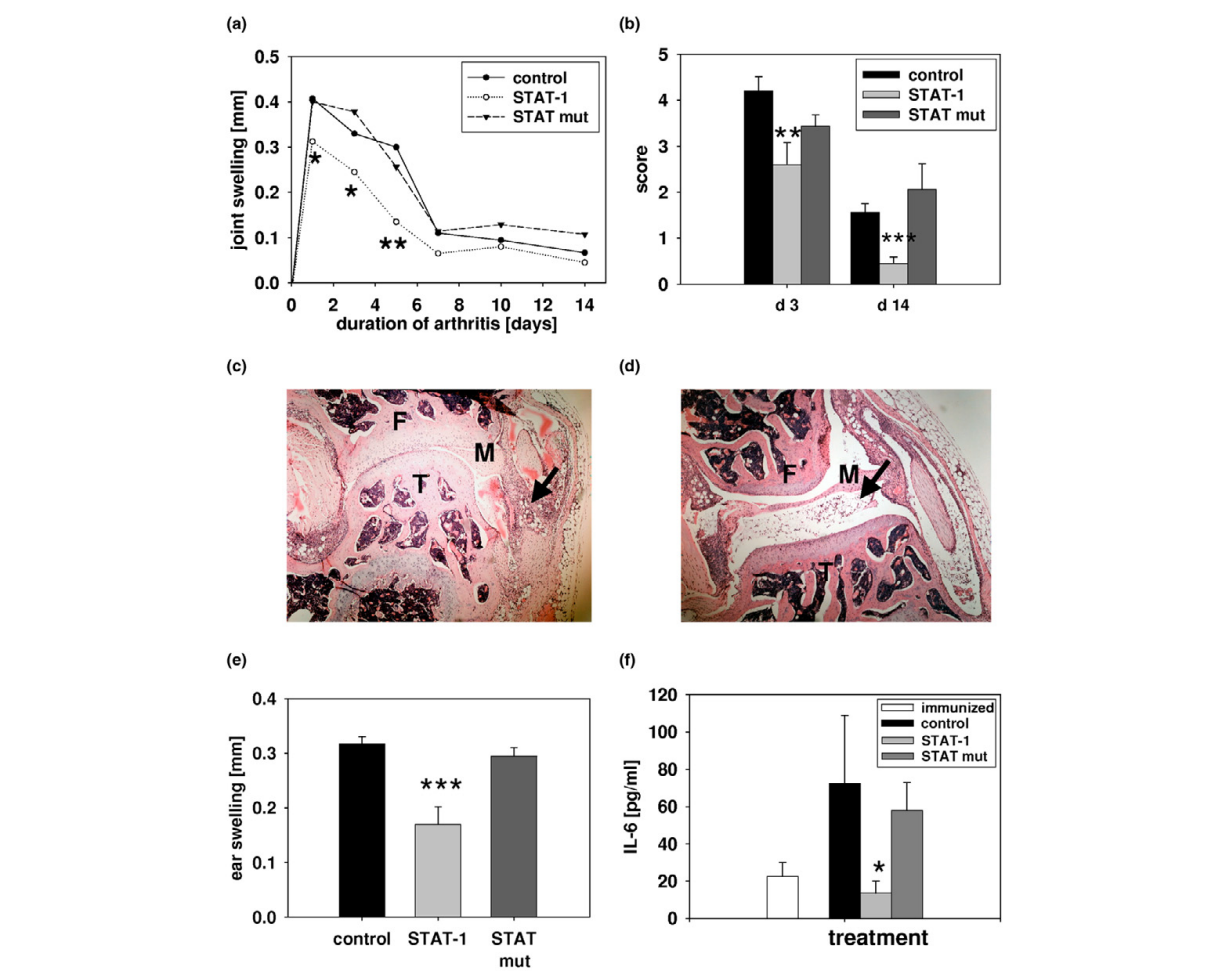
Specificity of the STAT-1 decoy oligodeoxynucleotide (ODN) treatment on the acute and chronic phase of antigen-induced arthritis (AIA). Arthritis was induced in the right knee joint at day 0, 4 h after application of the ODN. **(a) **Joint swelling after treatment with STAT-1 decoy ODN (STAT-1) and mutant control ODN (STAT mut) (all ODNs 0.25 nmol per knee; n = 8 to 10) in comparison to buffer-treated mice with AIA (control, n = 8 to 10). Joint swelling was assessed as the difference between right (arthritic) and left (untreated) knee joint diameter. **(b) **Total arthritis score after histological evaluation. Hematoxylin-eosin (HE) stained knee sections were evaluated blindly by grading of inflammation (cell exudate or infiltrate, hyperplasia) and joint destruction (necrosis, erosion and pannus formation). Total arthritis score is defined as the sum of the inflammation and destruction scores. Effects of STAT-1 decoy ODN (STAT-1) in comparison to mutant control ODN (STAT mut) (all ODNs 0.25 nmol per knee joint) on the acute phase (3 days after arthritis induction, d 3; n = 8 to 10) or on the chronic phase of AIA (14 days after arthritis induction, d 14; n = 8 to 10). **(c,d) **HE-stained sections of arthritic joints 3 days after arthritis induction. Knee joints of STAT-1 decoy ODN-treated mice **(d) **were less inflamed than joints of buffer-treated control animals **(c) **(see arrows). F, femur; T, tibia; M, meniscus. **(e) **Delayed-type hypersensitivity (DTH) reaction on day 7 after arthritis induction. Swelling was expressed as the difference (mm) between the ear thickness before and after antigen injection. The DTH reaction was also diminished in STAT-1 decoy ODN-treated mice, suggesting the involvement of STAT-dependent Tcell activation, whereas the STAT mutant ODN did not display such an effect **(f) **Effects on the serum levels of IL-6 at day 3 of AIA mice treated with the STAT-1 decoy ODN (STAT-1) or the STAT mutant ODN (STAT mut) (n = 8 to 10). Serum from immunized mice (day 0) served as reference. *P ≤ 0.05, **P ≤ 0.01, ***P ≤ 0.001 versus buffer-treated AIA control.

The STAT-1 decoy ODN had a clear therapeutic effect on AIA in C57BL/6 mice when injected intra-articularly 4 hours prior to induction of arthritis. The inhibitory effect of the STAT-1 decoy ODN was dose-dependent, achieving a significant reduction of joint swelling at a dose of 0.25 nmol per knee joint (Figure 1a). The beneficial effect of the STAT-1 decoy ODN on histology was also dose-dependent (Figure 1b). The evaluation of knee joint sections revealed a significant suppression of inflammation and cartilage destruction (Figure 1b). Knee joints of STAT-1 decoy ODN-treated animals were less inflamed than joints of untreated animals (Figure 2c,d). Whereas joint swelling was significantly decreased in the acute phase (days 1 to 5) after STAT-1 decoy ODN treatment, the mutant control ODN had no effect (Figure 2a). The mutant control ODN also had no significant effect on the histological arthritis score (Figure 2b), either in the acute phase (day 3) or the chronic phase (day 14) of AIA, further corroborating the specificity of the decoy ODN approach. In contrast to the mutant form, treatment with specific STAT-1 decoy ODN resulted in a significant reduction of the total arthritis score in both investigated phases (Figure 2b). Moreover, the DTH reaction was also diminished in STAT-1 decoy ODN-treated mice, suggesting the involvement of STAT-dependent Tcell activation, whereas the mutant control ODN did not display such an effect (Figure 2e).

### Effect of STAT-1 decoy oligodeoxynucleotide on cytokines in serum

Pro-inflammatory cytokines such as IL-1β, IL-6 and TNF-α are upregulated in RA as well as in AIA [20]. In sera, IL-6 was elevated in the acute phase (day 3) of AIA in comparison to non-arthritic, immunized animals (day 0 of AIA), and this effect was suppressed by the STAT-1 decoy ODN treatment (Figure 2f), while IL-1β and TNF-α levels were beneath the detection limit (data not shown). In the chronic phase of AIA, IL-6 decreased and was also below the detection limit in both STAT-1 decoy ODN-treated and control AIA mice (data not shown).

### Effect of STAT-1 decoy oligodeoxynucleotide on humoral immune responses

**Figure 3:**
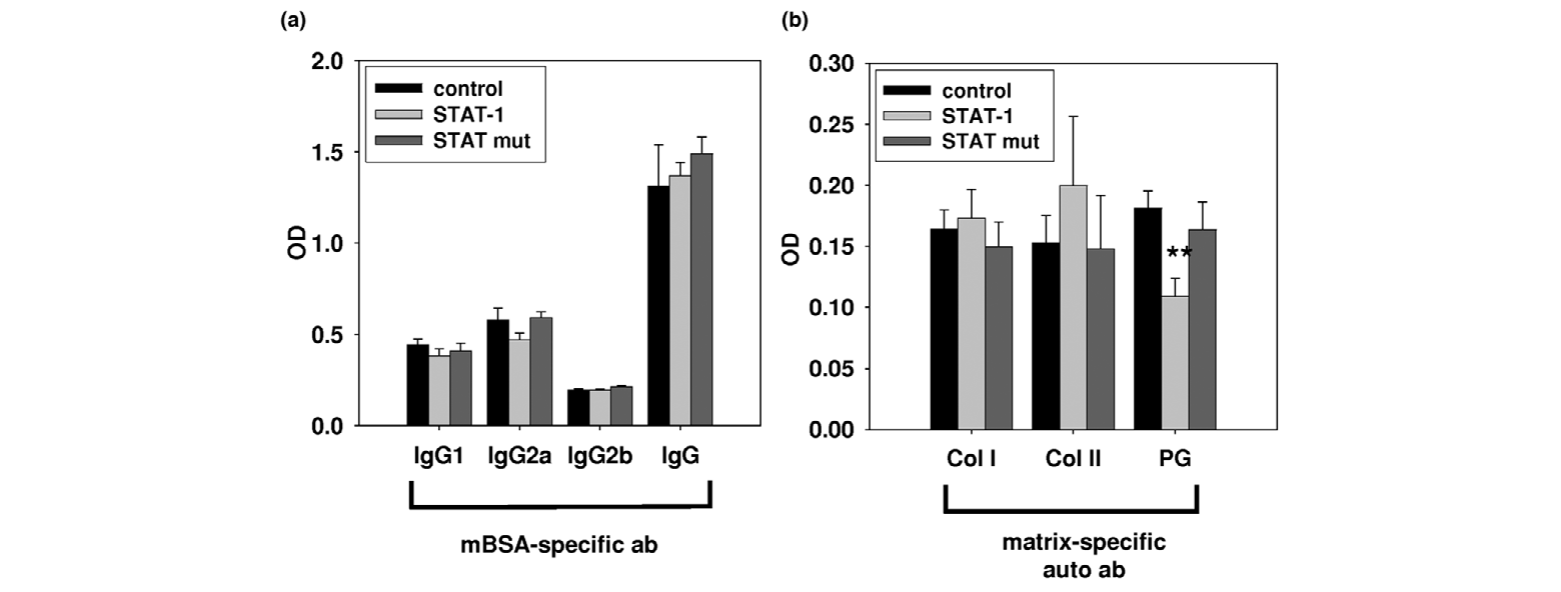
Effects of STAT-1 decoy oligodeoxynucleotide (ODN) treatment on the serum levels of antibodies (ab). **(a) **Methylated bovine serum albumin(mBSA)-specific IgG isotypes and **(b) **autoantigen specific IgGs (auto ab) against collagen type I (Col I), collagen type II (Col II) and proteoglycan (PG) in the serum of STAT-1 or STAT mutant (STAT mut) decoy ODN-treated mice 14 days after arthritis induction (n = 8 to 10). **P ≤ 0.01 versus buffer-treated AIA control.

In AIA, the serum levels of immunoglobulins against the antigen mBSA and the auto-antigens collagen type I, collagen type II and cartilage proteoglycans are elevated. All the studied antibodies (total IgG, IgG1, IgG2a, IgG2b) with specificity against mBSA were not altered by STAT-1 decoy ODN treatment (Figure 3a). Collagen type I and type II specific IgGs were also not changed, although proteoglycan-specific IgGs were significantly diminished by treatment with the STAT-1 decoy ODN (Figure 3b).

### *In vivo *distribution of internalised decoy oligodeoxynucleotide after injection into the joint space

**Figure 4:**
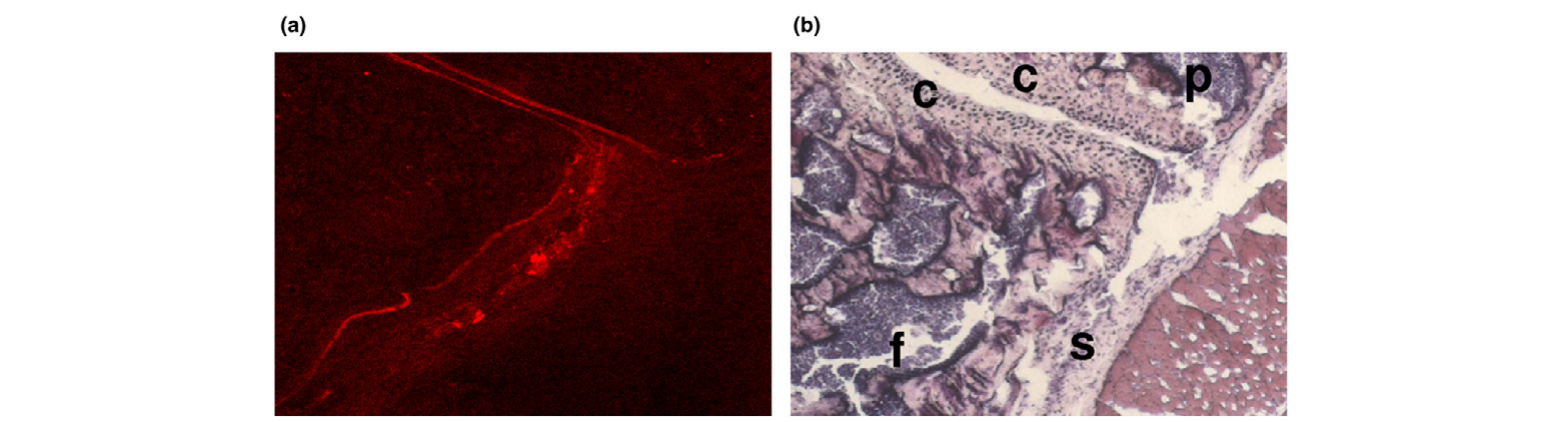
Detection of Texas Red-labelled STAT-1 consensus decoy oligodeoxynucleotide (ODN) after intra-articular administration. **(a) **Mice were sacrificed 1 h post injection. Internalisation of the labelled STAT-1 decoy ODN as judged by laser scanning microscopy was observed in the synovium region. Untreated control knees did not show any fluorescence signal (not shown). **(b) **Hematoxylin-eosin stained section of the same region in the knee joint: joint space between patella (p) and femur (f); articular cartilage (c); synovium (s).

To visualize the uptake of the decoy ODN *in vivo*, Texas Red-labelled ODN was administered intra-articularly and its distribution was observed in native histological sections 1, 3 and 6 h after injection. Using laser scanning microscopy, uptake of the STAT-1 decoy ODN as early as 1 h after injection could be observed. Strongest fluorescence was seen in the synovial lining layer (Figure 4a). The same cellular distribution could be seen 3 and 6 h after injection (not shown) but with weaker intensity because the fluorescence signal was quenched, presumably by intra-articular/intracellular cleaving. The biological effect of the unlabelled STAT-1 decoy ODN appears to be much longer and starts shortly after the uptake into the treated cells as shown by Quarcoo *et al*. [31] in a murine asthma model.

### STAT-1 decoy oligodeoxynucleotide efficacy in synoviocytes and macrophages *in vitro*

**Figure 5:**
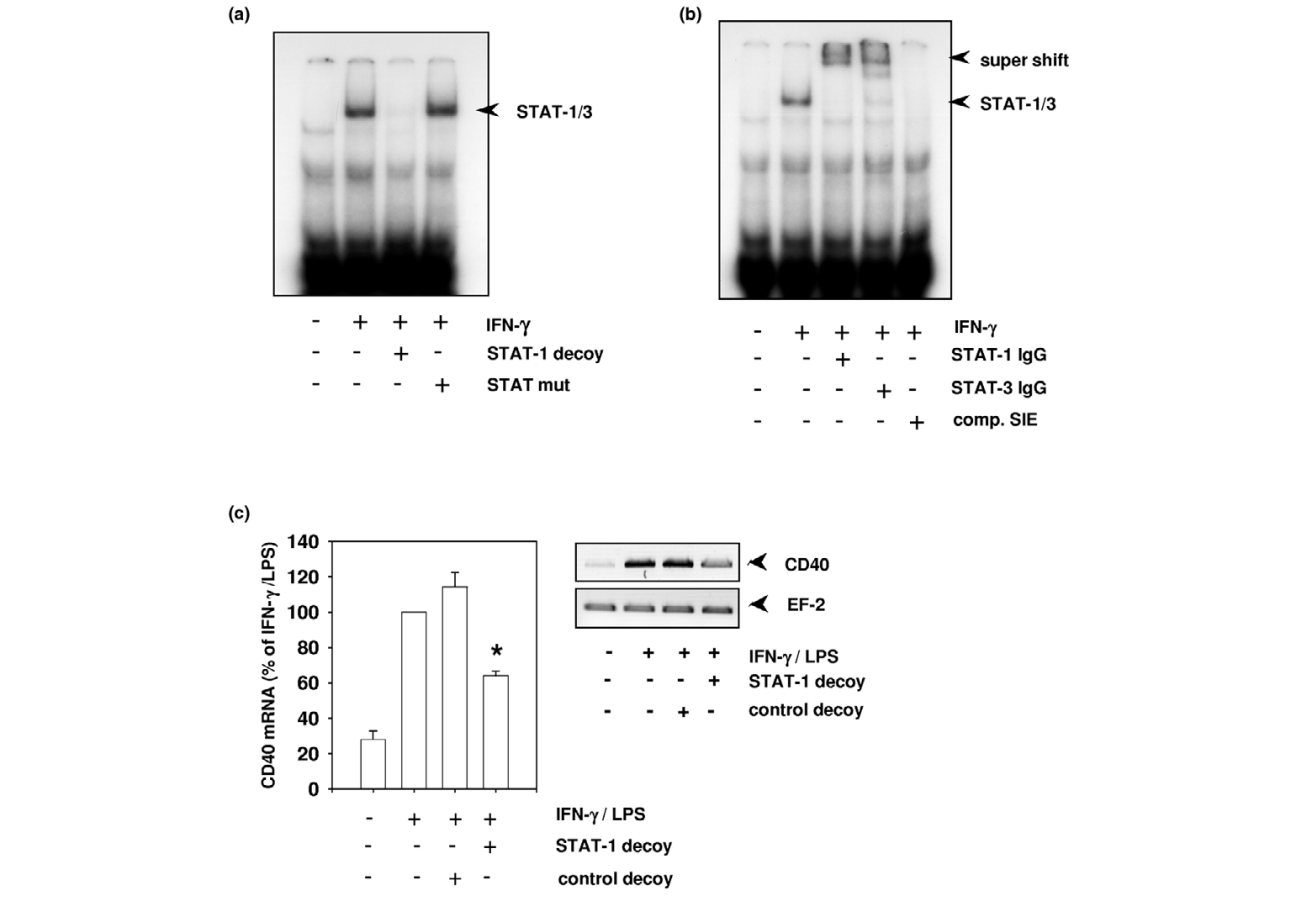
Efficacy of the STAT-1 decoy oligodeoxynucleotide (ODN) treatment in synoviocytes and macrophages *in vitro*. **(a) **Electrophoretic mobility shift analysis with extracts from synoviocytes stimulated by IFN-γ (250 U/ml, 30 minutes) and pre-treated (4 h before stimulation) with STAT-1 decoy ODN or STAT mutant ODN (STAT mut; 10 μM each). **(b) **Activation of STAT-1 and STAT-3 in IFN-γ-stimulated synoviocytes (250 U/ml IFN-γ, 30 minutes) was confirmed by gel shift supershift analysis with specific IgG antibodies. In addition, a 100-fold excess of the unlabelled compensatory sis-inducible element (comp. SIE) gel shift ODN abolished the STAT band, thus verifying the specificity of the detected band. **(c) **Statistical summary of the effect of STAT-1 decoy ODN (10 μM, 4 h pre-incubation) on CD40 mRNA expression in LPS/IFN-γ (1 μg/ml LPS plus 500 U/ml INF-γ)-stimulated RAW-264.7 macrophages after 12 h (n = 3 to 4, *P ≤ 0.05 versus LPS/IFN-γ). The mutant control ODN had no effect on CD40 expression. The insert shows a representative RT-PCR analysis (amplification of elongation factor-2 (EF-2) cDNA served as an internal standard).

Electrophoretic mobility shift analysis for SIE binding activity of nuclear extracts from IFN-γ-stimulated synoviocytes revealed increased STAT-1 and STAT-3 activation in these cells (Figure 5a,b), which was confirmed by supershift analyses using specific antibodies (Figure 5b). Moreover, the STAT-1 decoy ODN prevented binding of STAT-1/STAT-3 heterodimers from IFN-γ-stimulated synoviocytes to the SIE gel shift ODN (Figure 5a). In contrast, the mutant control ODN did not affect binding to the radioactive labelled SIE gel shift ODN by activated STAT-1 and STAT-3 under these conditions (Figure 5a). Moreover, we performed additional gel shift experiments using STAT-1 (Santa Cruz, sc-2573) and STAT-3 (Santa Cruz, sc-2571) specific gel shift ODNs, which gave comparable results (not shown). Taken together, these results clearly demonstrate that activated STAT-1 and STAT-3 can bind to the therapeutic decoy ODN, either as homodimers or as heterodimers.

To check if STAT-1 signalling may have a direct effect on CD40 expression in macrophages, RAW 264.7 cells were stimulated with LPS plus IFN-γ *in vitro*, resulting in an upregulation of CD40 expression as shown by RT-PCR analysis (Figure 5c). When the STAT-1 decoy ODN was added to the cultured cells prior to LPS/IFN-γ stimulation, a significant reduction of CD40 mRNA was observed, suggesting the involvement of STAT-1 signalling in CD40 expression in macrophages. In contrast, the mutant control ODN had no effect on CD40 expression in these cells (Figure 5c).

### STAT-1, STAT-3, IRF-1 and CD40 expression during the time course of antigen-induced arthritis

**Figure 6:**
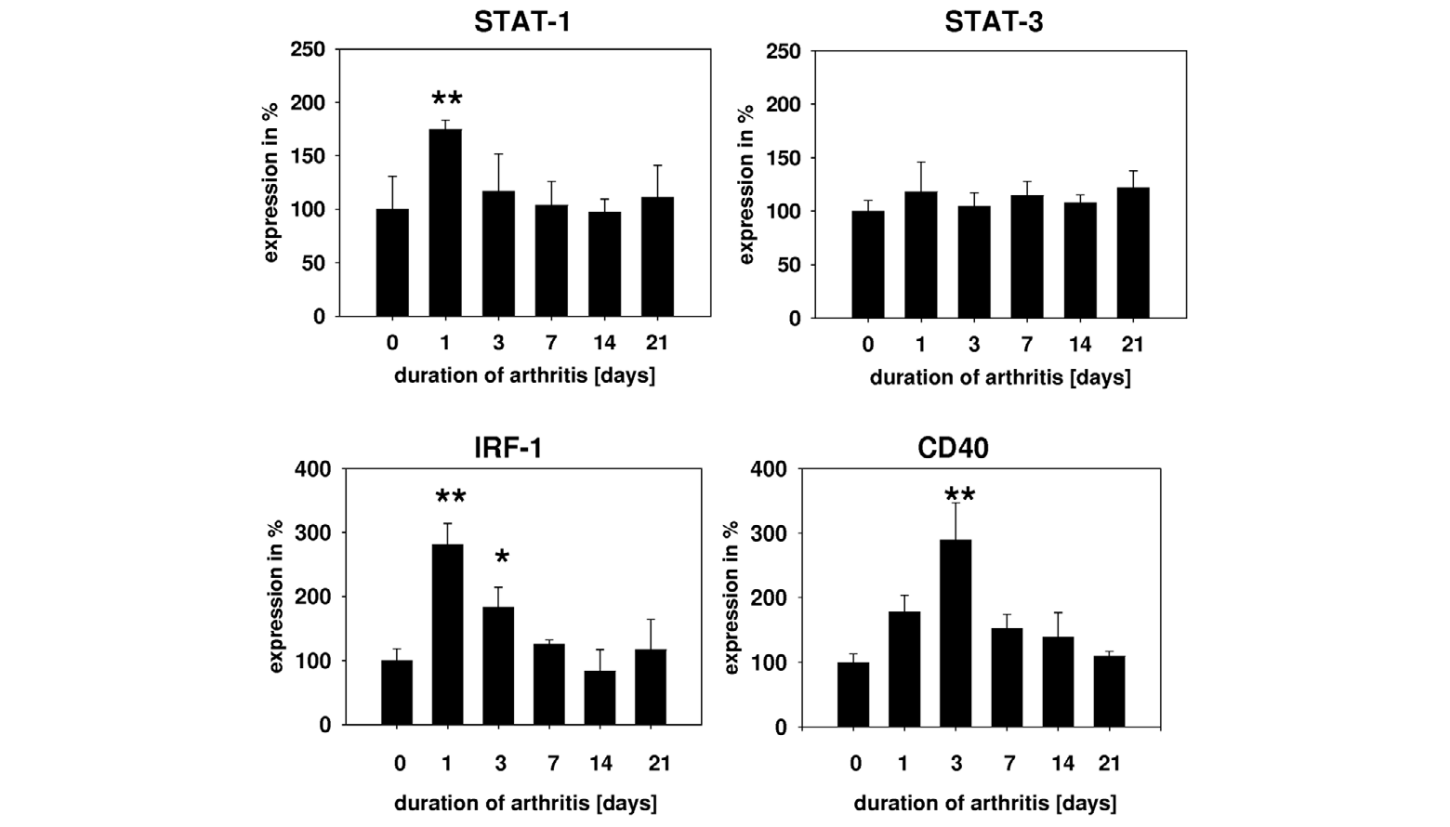
mRNA expression of STAT-1, STAT-3, interferon regulatory factor (IRF)-1 and CD40 in murine arthritic knee joints. Total RNA was isolated from joint capsules including the synovial tissue before (day 0) and after (day 1 to day 21) arthritis induction (n = 5 for day 0, 1, 3 and 7; n = 4 for day 14 and 21). After reverse transcription, mRNA expression of STAT-1, STAT-3, IRF-1 and CD40 was measured by real time PCR. The house keeping genes encoding β-actin (for STAT-1 and STAT-3) and GAPDH (for IRF-1 and CD40) were used for normalization of expression. Mean of day 0 expression was set at 100%. *P ≤ 0.05, **P ≤ 0.01 versus day 0.

To confirm that STAT-1 plays a main role in the AIA model, mRNA was isolated from joint capsules including the synovial tissue at different time points of arthritis (days 1 to 21) as well as from non-arthritic, immunized mice (day 0). The mRNA expression was analysed by real time PCR for STAT-1, STAT-3, IRF-1, and CD40. In comparison to control mice (day 0), expression of STAT-1 was significantly elevated at day 1 of arthritis (Figure 6). STAT-3 expression showed no changes during this time course (Figure 6). Additionally STAT-1-dependent IRF-1 expression was significantly upregulated at day 1 and 3 of AIA (Figure 6). CD40 was also differentially expressed during the course of AIA and significantly upregulated at day 3 (Figure 6).

### Anti-CD40L treatment led to similar attenuation of arthritis as STAT-1 decoy oligodeoxynucleotide treatment

**Figure 7:**
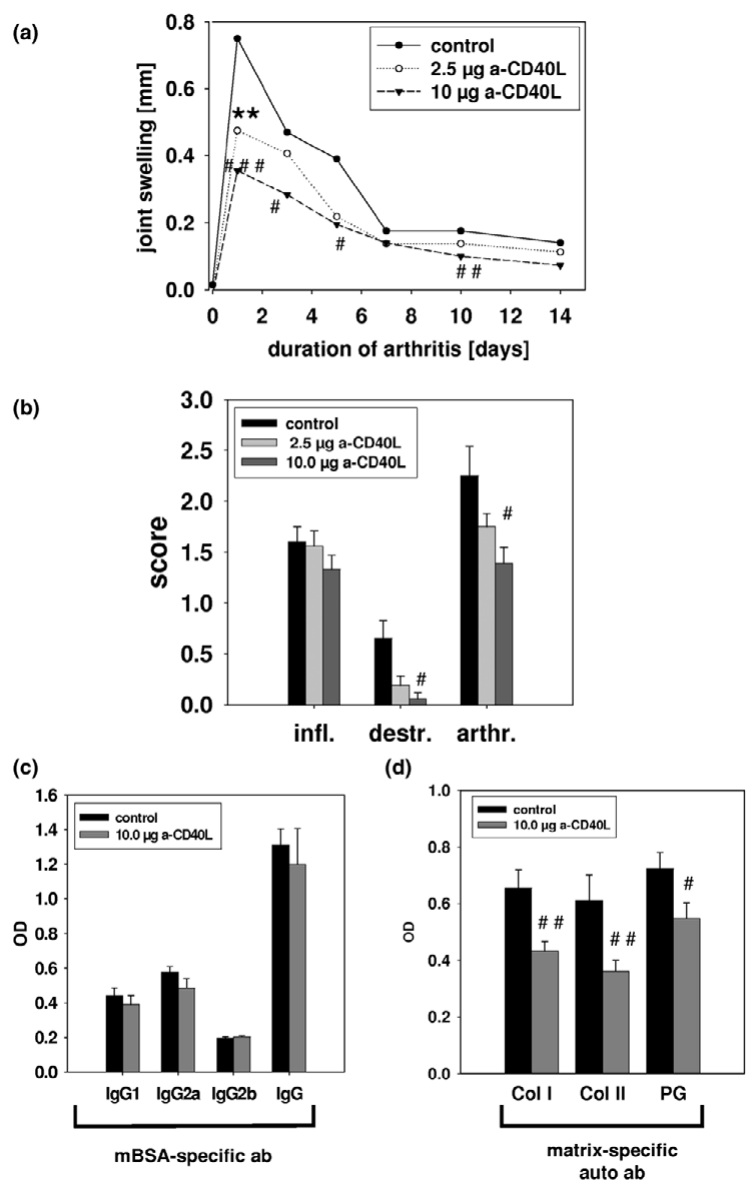
Dose-dependent effects of anti-CD40L monoclonal antibody (a-CD40L) treatment on antigen-induced arthritis. Effects on **(a) **knee joint swelling and **(b) **histology (n = 9). Hematoxylin-eosin stained knee sections were evaluated as described in Figure 1. **(c,d) **Effects on the serum levels of methylated bovine serum albumin (mBSA)-specific IgG isotypes and autoantigen-specific IgGs (against collagen type I (Col I), collagen type II (Col II) and proteoglycan (PG));**P ≤ 0.01, (2.5 μg a-CD40L versus buffer-treated AIA control). ^#^*P *≤ 0.05, ^##^*P *≤ 0.01, (10 μg a-CD40L versus buffer-treated AIA control). Infl., inflammation; destr., destruction; arthr., total arthritis score.

To ascertain if STAT-1-induced CD40 expression may play a role in the development of AIA, experiments with anti-CD40L (anti-CD154) monoclonal antibodies were performed in parallel. Joint swelling (Figure 7a) and histological evaluation (Figure 7b) disclosed that inhibition of CD40-CD40L by antagonizing CD40L can ameliorate the disease, supporting the notion of an involvement of CD40L-dependent T cell activation. Treatment with the anti-CD40L monoclonal antibodies did not affect the mBSA-specific IgGs either (Figure 7c), but reduced the serum levels of IgG against collagen type I and type II as well as cartilage proteoglycans (Figure 7d).

## Discussion

RA is a systemic disease accompanied by high inflammation of affected joints. Th cells and their cytokines are assumed to play a major role in driving inflammation in RA and, thus, in inducing destruction processes. Most of the Th cell cytokines exert their effects via the JAK/STAT pathways. IFN-γ is the strongest activator of STAT-1, however, IL-6, IL-10, and IFN-β can also contribute to synovial STAT-1 activation. Thus STAT-1 activation as well as increased expression of STAT-1-dependent genes were found in a subgroup of patients with RA accompanied by a massive infiltration of inflammatory cells [8]. In addition, STAT-1 expression has been shown in T and B lymphocytes in focal inflammatory infiltrates, in synovial fibroblasts and synovial macrophages of the intimal lining layer [10]. Collectively, these findings suggest that STAT-1 drives pro-inflammatory gene expression in RA patients and, thus, may represent a novel therapeutic target for this disease.

The application of decoy ODNs might represent a novel promising approach to inhibit RA-related changes in gene expression. Decoy ODNs are small double-stranded DNA molecules that enter target cells without auxiliary means. They are protected against degradation by the introduction of phosphorothioate instead of phosphodiester bonds. They neutralize their target transcription factor, thus preventing its binding to specific regulatory sequences in the promoter of its target genes and thereby effectively inhibiting its expression. Using murine AIA as a Th1-mediated inflammation model, we investigated the therapeutic effects of a STAT-1 decoy ODN with a binding site similar to the GAS. Murine AIA is a well established experimental arthritis model showing homologies to human RA in terms of histopathology and responses to anti-inflammatory and immunomodulatory therapies. AIA can be divided into two phases. The first phase, the acute stage, is characterized by joint swelling and infiltration of different cells of the immune system. Pannus formation and matrix degradation are hallmarks of the subsequent chronic phase.

The STAT-1 decoy ODN was injected intra-articularly four hours prior to arthritis induction to allow sufficient cellular uptake and maximize efficacy at the time of induction. In the AIA model, administration of the STAT-1 decoy ODN reduced typical symptoms such as joint swelling, the DTH reaction and histopathological signs of arthritis. Clinical benefits may be attributed to the inhibition of the binding of activated STAT-1 proteins to specific promoter sequences of AIA-relevant genes as the mutant control ODN had no significant effects.

The role and importance of STAT-1 in synovitis is still a subject of controversial discussion [7]. Indeed, STAT-1 can play both a pathogenic and a protective role in RA synovitis, depending on the cell type and possibly on the stage of disease. Studies using STAT-1-deficient mice and cells have shown that STAT-1 mediates the antiviral and immune/inflammatory effects of IFNs, and that it mediates the induction of immune effectors and inflammatory genes, such as HLA, costimulatory molecules, chemokines, complement, IRF-1, inducible nitric oxide synthase and FcRI genes [32-35]. Alternatively, STAT-1 induces growth arrest and promotes apoptosis in several cell types, including lymphocytes and synovial fibroblasts [36-39]. These functions suggest a protective role for STAT-1 in arthritis, and this role is supported by elevated expression of the STAT-1 pro-apoptotic target gene caspase-1 in RA synovium [8]. Moreover, in STAT-1-deficient mice, increased joint inflammation in zymosan-induced arthritis has been observed [40] as well as increased susceptibility to experimental autoimmune encephalomyelitis; in addition, these mice overexpressed the myeline basic protein-specific T cell receptor [41]. The authors suggest that the increased susceptibility to experimental autoimmune encephalomyelitis is related to impaired function of regulatory T cells, which is STAT-1-dependent. Targeting of STAT-1 may thus even promote disease pathology, at least in some cells. We concluded from these observations together with our own experimental results that the STAT-1 decoy ODN exerted a therapeutic effect in our AIA mouse model because it targeted non-proliferating cells, such as synovial macrophages. By using Texas Red-labelled ODN it could be demonstrated that it was absorbed well and mainly localized to sites of the synovium. Additionally, our *in vitro *experiments with preparations of synoviocytes have shown that the applied STAT-1 decoy ODN, but not the mutant form, binds to STAT-1/STAT-3 heterodimers. We conclude, therefore, that the binding of activated STAT-1 and/or STAT-3 to certain target gene promoters in macrophages and synoviocytes can be blocked by treatment with the administered STAT-1 decoy ODN *in vitro *and *in vivo*.

Macrophages play a central role in the pathogenesis of RA and also in the development of AIA. Stimulation of human monocytes with IFN-γ activates STAT-1 through phosphorylation, resulting in an increased expression of STAT-1 at mRNA and protein level [15]. In contrast, phosphorylation and expression of STAT-3 was only marginally affected by IFN-γ treatment in these cells. IFN-γ stimulation of RAW264.7 macrophages caused both phosphorylation and an increase of STAT-1 mRNA expression [42]. In our experiments, elevated mRNA levels for only STAT-1 but not for STAT-3 were detectable in joint capsules containing the synovial tissue in the course of AIA. In addition, we have recently shown that the local concentration of IFN-γ rises in the arthritic knee joints on day 1 after induction of AIA [43], hence coinciding with the observed increase in STAT-1 expression in this study. Moreover, IRF-1 is a classic STAT-1-dependent, IFN-γ-induced target gene, as shown with STAT-1-deficient cells [34]. The promoter of IRF-1 contains several GAS elements [18,44,45]. In our hands, expression of IRF-1 at mRNA level was also significantly elevated at day 1 and 3 of the acute phase of AIA. These data imply that activation of the STAT-1 pathway plays an important role in our Th1 cell-mediated arthritis model.

Th1 cell function is already an essential pathogenic component during the acute phase of AIA [26]. Anti-CD4 monoclonal antibody treatment led to a lower expression of IFN-γ and IL-2 in spleen cells but did not affect the secretion of IL-4 and IL-10. In addition, the concentration of IL-6 in the serum and its secretion by macrophages were also decreased [46]. IFN-γ is a potent inducer of CD40 expression in macrophages because phosphorylated STAT-1 binds to the GAS element of the CD40 promoter [47,48]. We confirmed this finding by blocking the CD40 expression in IFN-γ/LPS-stimulated STAT-1 decoy ODN-treated RAW264.7 macrophages. Further, supporting the pivotal role of macrophages in our AIA model, we observed a reduction in the DTH response in STAT-1 decoy ODN-treated animals in comparison to the mutant control ODN-treated group. The T cells responsible for DTH are members of the CD4^+ ^subset. Macrophages are the effector cells in the DTH response. When the DTH response is reduced then the macrophage response must be impaired too. In fact, in mice a reduction of CD40 expression seems to be responsible for the reduced DTH response in an IL-12-dependent way [49].

It was demonstrated recently that freshly isolated synovial cells from RA patients express CD40, whereas synovial T cells express CD40L (CD154) [50]. As CD40-CD40L interactions have been implicated in arthritis [47,51] a reduction in CD40 expression due to STAT-1 decoy ODN application may explain the attenuated arthritic symptoms in our AIA model. The main effect of the STAT-1 decoy ODN observed in AIA might be due to the impaired CD40-dependent activation of macrophages in the synovial membrane after the Th1 cell-derived IFN-γ and CD40L stimulation. We arrived at this conclusion irrespective of STAT-3 supershift in IFN-γ-stimulated synoviocytes and irrespective of the possibility that other cell types in the knee joints may be influenced by the STAT-1 decoy ODN. This notion is supported by the fact that the anti-CD40L antibody approach also resulted in a profound reduction of arthritis in our model. The observed decrease in the arthritis score and in the disease symptoms were comparable in the anti-CD40L and STAT-1 decoy ODN arm of the study. The role of macrophages in STAT-1-mediated effects is also supported by the observation that STAT-1 phosphorylation in murine zymosan-induced arthritis occurred first after infiltration of mononuclear cells into the synovium [40]. Moreover, IFN-γ has been shown to markedly upregulate IL-12 production by CD68^+ ^synovial cells through not only CD40/CD40L-dependent, but also independent, pathways [52]. Macrophages from synovial fluids of RA patients can differentiate *in vitro *into dendritic cells, the main producers of IL-12 [53].

Furthermore, our hypothesis is supported by the demonstration that CD14^+ ^synovial cells interact with fibroblast-like synoviocytes after CD40L stimulation via the soluble mediators TNF-α, IL-1α and IL-1β. These cytokines induce the production of downstream cytokines such as IL-6 in fibroblast-like cells [54]. A further increase of CD40L-dependent IL-6 secretion by fibroblast-like synoviocytes could be observed after treatment of CD14^+ ^synoviocytes with IFN-γ [54]. As fibroblast-like synoviocytes themselves expressed CD40 and responded to CD40L to produce IL-6, IL-8 and MCP-1(monocyte chemoattractant protein-1) [54-56], these cells would be activated both directly and indirectly by CD40L-expressing T cells in the synovial tissue of RA patients. Congruently, we observed in our experiments decreased levels of IL-6 in the serum of STAT-1 decoy ODN treated mice.

Despite the primary therapeutic mechanism on synovial macrophages expressing CD40 in our experiments and the recently described pro-apoptotic effect of STAT-1 in fibroblasts [39], a direct therapeutic effect of STAT-1 decoy ODN on synovial fibroblasts via influencing CD40 expression cannot be excluded. Nevertheless, the reduction of IL-6 expression after STAT-1 decoy ODN treatment will cause a decreased activation of STAT-3 in synovial fibroblasts, where it promotes cell survival and proliferation [39].

## Conclusions

Our experiments demonstrate that a single local application of a decoy ODN neutralizing the transcription factor STAT-1 effectively inhibits antigen-induced arthritis, most likely through attenuating the enhanced expression of CD40 and other effector molecules in macrophages. These data suggest that STAT-1 might play a pivotal role in the development of arthritis and, therefore, might represent a potential novel target for the treatment of human RA.

## Abbreviations

AIA = antigen-induced arthritis; AP = alkaline phosphatase; bp = base pairs; CD40L = CD40 ligand (CD154); DMEM = Dulbecco's modified Eagle's medium; DTH = delayed-type hypersensitivity; ELISA = enzyme-linked immunosorbent assay; EMSA = electrophoretic mobility shift analysis; FCS = fetal calf serum; GAS = γ-activated sequence; IFN = interferon; IL = interleukin; IRF = interferon regulatory factor; JAK = janus kinase; LPS = lipopolysaccharide; mBSA = methylated bovine serum albumin; ODN = oligodeoxynucleotide; PAGE= polyacrylamide gel elecrtrophoresis; RA = rheumatoid arthritis; RT-PCR = reverse transcription polymerase chain reaction; SIE = sis-inducible element; STAT = signal transducer and activator of transcription; Th = T-helper; TNF = tumour necrosis factor.

## Competing interests

The authors declare that they have no competing interests.

## Authors' contributions

MHu and US contributed equally to this work. MHu and RS performed and assessed the animal studies. MHu performed the ELISAs, the *in vitro *cell culture experiments and wrote the manuscript. US assessed and analyzed the mRNA expression data of STAT-1 and STAT-3, and participated in the layout, writing, and finalization of the manuscript. PKK, SH and MG did the histological evaluation. AHW performed the EMSAs, the CD40 and IRF-1 expression analysis and provided the STAT-1 decoy ODN. KT did the statistical analysis. MHe and RB designed and coordinated the project. All authors read and approved the final manuscript.
